# The Immune Epitope Database: How Data Are Entered and Retrieved

**DOI:** 10.1155/2017/5974574

**Published:** 2017-05-29

**Authors:** Ward Fleri, Kerrie Vaughan, Nima Salimi, Randi Vita, Bjoern Peters, Alessandro Sette

**Affiliations:** Division of Vaccine Discovery, La Jolla Institute for Allergy and Immunology, 9420 Athena Circle, La Jolla, CA 92037, USA

## Abstract

Easy access to a vast collection of experimental data on immune epitopes can greatly facilitate the development of therapeutics and vaccines. The Immune Epitope Database and Analysis Resource (IEDB) was developed to provide such a resource as a free service to the biomedical research community. The IEDB contains epitope and assay information related to infectious diseases, autoimmune diseases, allergic diseases, and transplant/alloantigens for humans, nonhuman primates, mice, and any other species studied. It contains T cell, B cell, MHC binding, and MHC ligand elution experiments. Its data are curated primarily from the published literature and also include direct submissions from researchers involved in epitope discovery. This article describes the process of capturing data from these sources and how the information is organized in the IEDB data. Different approaches for querying the data are then presented, using the home page search interface and the various specialized search interfaces. Specific examples covering diverse applications of interest are given to highlight the power and functionality of the IEDB.

## 1. A High-Level Overview of the IEDB

The Immune Epitope Database (IEDB) is a free online resource that catalogs and makes accessible to the scientific community epitope-related data derived from allergic diseases, infectious diseases, apart from HIV which is captured separately in the Los Alamos HIV database [1], autoimmune diseases, and diseases associated with transplantation and alloantigens. The IEDB [2] contains both T cell and B cell epitopes, as well as MHC ligand data. The epitopes are derived from humans, nonhuman primates, mice, and all other studied hosts.

The curation of scientific literature started in 2004, requiring the curation of past and current relevant epitope literature in available peer-reviewed journals [3, 4]. As the IEDB evolved, it has been necessary to change how biological concepts are captured in order to maximize accuracy. In addition, automated validation is continuously added. Consequently, there is a significant ongoing “recuration” effort of revising existing entries to improve data quality and consistency. The IEDB is now current with the published literature, and targeted PubMed queries are run biweekly, with the goal of making the data available in the IEDB within eight weeks of publication.

As of November 2016, the IEDB data were derived from over 18,000 papers. In addition, the IEDB contains nearly 300 submissions (corresponding to approximately 20% of the total data) from several NIH-funded large-scale epitope discovery programs and from researchers that directly approach the IEDB to deposit their data, including negative data, which might typically appear in supplemental tables or might not be published at all. Slightly more than half of the references in the IEDB relate to infectious diseases and about a quarter relate to autoimmune diseases. The remainder includes allergy, transplant, and other categories. Because the data in the IEDB reside in the public domain, researchers can freely access, analyze, and publish works using these data.

When initially designing the IEDB, we realized that different researchers had different views on what should be included in an epitope database, based largely on their area of research. While each scientist's specific interests and epitope definitions might vary, ultimately every epitope is defined by an experiment or specific assay. To provide a general yet accurate epitope database, we utilized an assay-centric design, capturing the experiments (assays) that characterize and define each epitope. This required translation of the data typically described in the methods and results sections of a scientific paper into a generic data structure in which epitope data are entered and stored in a format that allows users to query for the characteristics of these epitopes.

The IEDB website has undergone two major revisions since its introduction in early 2006, each improving its features and usability [2, 5]. The current IEDB 3.0, deployed in February 2015, incorporated feedback from immunologists and bioinformaticians, collected at user workshops, help desk requests, and user observation sessions, to make searching the database more intuitive and to deliver results in a more useful format. The home page prominently features the query interface, which contains the fields that address over 80% of the typical queries. The query concept is similar to a travel website where users specify basic information and can then filter or refine the results with additional fields. In the following sections, we will present a detailed account of the IEDB query and reporting and also provide several specific examples.

## 2. Which Data Are Included in the IEDB?

The IEDB does not capture predicted/inferred data or reviews. All data come from empirically derived assays reflecting the interaction of an adaptive immune receptor with an epitope. There are four categories of assays—T cell, B cell (including soluble antibodies), MHC binding, and MHC ligand elution.

Data are captured in accordance with clearly established criteria [5]. Linear peptides cannot exceed 50 amino acids in length and must be tested as either an immunogen or an antigen. We capture discontinuous epitopes where they show that specific residues are important for antibody recognition of an antigen, usually through mutation, or from crystal structures of B cell receptor and peptide. The database also includes nonpeptidic epitopes. These include carbohydrates, lipids, chemicals, metals, and drugs. In addition, for all data there is a minimal set of information required. For linear peptides, the amino acid sequence must be specified, and for discontinuous epitopes, the amino acid and their respective position must be specified (e.g., G104, G106, L107, and W231). In all cases, it must be possible to determine whether the outcome was positive or negative. In the case of T and B cell assays, a host (e.g., human or mouse) must be specified.

Because the IEDB is fundamentally a database of experiments, each paper or reference to be included must describe at least one epitope, and each epitope must be described in one or more experiments. Up to 400 data fields are used to capture all the details of these experiments. Simplifying the curation process and ensuring accuracy and consistency is largely addressed by the use of external resources and ontologies [6].

The goal of the IEDB is to allow a global query of all data from the scientific literature and direct submission. Individual epitopes can be tested in multiple references, in more than one host and under different conditions. If an epitope has been studied in multiple publications, users can gather all the cumulative data from the IEDB and draw their own conclusions regarding its utility for their application. The same epitope might be tested in different assay types or may be repeatedly tested in the same experimental setup by different groups. In some cases, the assay may be positive and in others negative and the user can examine the experimental details captured therein to critically evaluate the available information.

## 3. How Are the Data Structured?

As stated above, each IEDB record must be associated with a clearly defined molecular structure. In addition, for peptidic epitopes, the author must provide a sequence and identify its protein source and organism source. While some authors specify a GenBank or UniProt identifier for each protein, most provide a protein name or abbreviation. Since protein nomenclature is highly variable and a search for protein names in GenBank can yield a large number of results, the protein source assigned to a given peptide by the IEDB must have a 100% BLAST match [7] with that sequence and must correspond to the protein that the author indicated.

Each peptidic epitope is also derived from an organism species, and authors may further specify a particular strain. The IEDB uses the NCBI taxonomy (https://www.ncbi.nlm.nih.gov/taxonomy) to formalize the organism assignment. The extensive taxonomy tree has been modified to only include species found in the IEDB and subsequently embedded in an organism finder, augmented with synonyms and auto-complete features to facilitate queries.

The curation of nonpeptidic structures within the IEDB conforms with the Chemical Entities of Biological Interest (ChEBI) database in the UK (https://www.ebi.ac.uk/chebi/) [8]. For this, a ChEBI curator with specific chemistry expertise assists in describing each chemical structure captured in the database. In the case of nonpeptidic epitopes, the ChEBI curator reads the paper, draws the structure, deposits it into the ChEBI database, and provides the ChEBI identifier, which is then used to curate all associated assays with that structure [9].

To describe the immunization processes, the NCBI taxonomy tree is again used to specify the host organism whose T cells or antibodies are being studied in experiment. The immunization process describes how the host became initially exposed or sensitized. For example, a mouse was injected with a protein, a human was given a vaccine, or a human contracted a specific disease. The Disease Ontology (DO) [10] is used to provide a standardized list of disease names and identifiers, which enables linkage of the IEDB data to other databases using the same identifiers. The use of external ontologies like DO provides useful information, such as definitions, synonyms, and relationships that the IEDB can leverage to enhance its information content.

The Ontology of Biomedical Investigation (OBI) [11], which gives a hierarchical tree structure of all assay types found in the literature, is used to capture the details of the experiment accurately and as completely as possible. The hierarchical tree structure allows users to search at many levels, for example, on all T cell assays, or specifically cytokine assays, or only IL-2 assays. As we encounter new assay types, we request OBI to add new terms to the ontology, which can then be used by our curators and end users.

MHC restriction is an important parameter related to T cell assays, as well as MHC binding and ligand elution assays. Because no MHC allele ontology existed, the IEDB created the MHC Restriction Ontology (MRO). MRO models the protein complex of the MHC as being composed of two different protein chains that are each encoded by different loci that come together to form a complex [12]. Users can search by locus, protein chain, or the whole MHC complex. In addition, haplotype information is included for mice.

## 4. The Curation Process

The literature curation process starts with the execution of a complex keyword query of PubMed to retrieve potentially relevant papers. An automated classifier [13–15] then assesses the likelihood that a paper contains relevant information and assigns each paper retrieved to a disease category. Curatability is then reviewed by a staff immunologist, and papers are assigned to the curation team. Individual papers are read, assessed for meeting strict inclusion criteria (http://curationwiki.iedb.org/wiki/index.php/IEDB_Inclusion_Criteria), and then entered into the database. About 30% of the papers do not meet the inclusion criteria and are marked therefore as uncuratable. Although the abstract may contain the requisite keywords, the actual article commonly might omit sequence information or might not actually be about epitopes. Articles about NK epitopes, epitope tags, superantigens, and antigen processing are also excluded.

Curators use a specialized internal application called a “finder” to assign a GenBank (https://www.ncbi.nlm.nih.gov/genbank/) or UniProt (http://www.uniprot.org/) identifier to each epitope based on 100% sequence identity. Finders are incorporated into the curation system and the external production system to facilitate input selections and the use of controlled vocabulary. They organize various query parameters, such as molecules and organisms, in a hierarchical tree fashion. As an example, the hemagglutinin protein from influenza A virus has more than 300 different GenBank entries that capture differences in the epitope sequences encountered in the literature, but these are presented by the finder as a single hemagglutinin entry.

**Figure 1:**
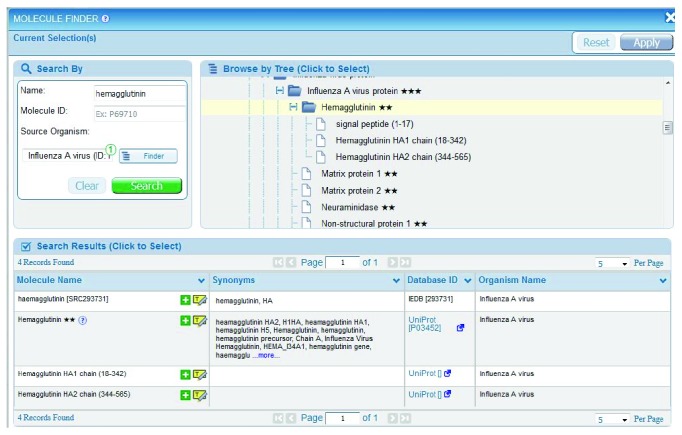
The Molecule Finder can be used to select the source antigen of the epitope. The yellow highlight icon in the bottom table highlights the selection in the tree in the upper right. The green “+” icon selects the molecule for the search criteria. The stars next to the molecule name indicate the quality and completeness of the associated reference proteome.

To avoid sorting through this many possible sequences of hemagglutinin when performing a query, the protein source data are organized using a UniProt reference proteome. UniProt provides a list of all the proteins for a given species that is used to group all the proteins used as epitope sources. Therefore, only the UniProt hemagglutinin is displayed in the finder (Figure 1). Stars (^∗∗^) are used to indicate the quality and completeness of the associated reference proteome for that organism. Within the finder application, a question mark icon provides a link that elaborates on the star grading system. UniProt also provides information on the processing of proteins into functional fragments. The IEDB search interface embeds these UniProt annotations to enable searches on processed fragments or on full-length proteins. Users can also query all influenza virus proteins by selecting a higher node in the finder tree. Similar finders with hierarchical trees are used throughout the query interface.

All curated papers undergo peer review to determine if the captured data are accurate. If necessary, authors are contacted for clarifications on the paper's content. New data are promoted on the IEDB website weekly, representing 15–20 new papers per week.

The papers themselves can differ greatly, given the variety of journals, authors, and diseases, making data consistency a challenge. To address these differences, a number of quality control measures have been implemented. Formal curation guidelines have been developed over the years as we encounter new concepts and techniques in the literature (http://curationwiki.iedb.org/wiki/index.php/Curation_Manual2.0) [16]. We also refer to immunological experts as needed, especially when modifications of how data are captured are proposed or new assays or immunological content is encountered. To assist and streamline the curation process, a web-based curation system has been developed with built-in validation logic. For example, when a curator inputs a certain value in the data field, input for other data fields are constrained to certain allowable values.

## 5. Direct Submission Processes

As mentioned above, the IEDB is populated with data from direct submissions in addition to literature curation. Direct submissions account for about 20% of the epitope records. The primary source of submissions has been the NIAID-funded large-scale T cell and antibody epitope discovery contracts and grants for infectious diseases, such as dengue virus, influenza virus, arenavirus, tuberculosis, and common allergens, although other researchers have deposited their epitopes as well.

There are three ways to submit data to the IEDB. The first is a wizard system that walks the submitter through a step-by-step process to enter data. This is ideal for new submitters who need to become familiar with the data fields and for those with only a few epitopes and assays. The second method utilizes Excel files that work as templates for different types of assays. This method accommodates larger data submissions and is the most commonly used. The third method uses XML files, which offer the greatest flexibility, but also require expertise in the use of XML and familiarity with the IEDB data structure. One of the IEDB curators specializes in assisting researchers in submitting data.

Just as all curated published literature has a PubMed ID that is captured in the IEDB, all data submissions are assigned a submission ID that can be used in publications and later referenced. The submissions are reviewed by curators to ensure completeness and accuracy, and the data validation rules are executed as done for data curated from literature. The data are not made publicly available until the author requests it, usually at the time that a corresponding manuscript is published. If a data submission is associated with a publication curated by the IEDB, the submission ID is linked to the PubMed ID.

## 6. IEDB Search Basics

The IEDB provides users with two basic options—the home page search and specialized searches. In both cases, special “finder” features assist in this process. Data field names are explicit and concise by necessity and may be unfamiliar to some users. For example, it is necessary to distinguish whether a protein is delivered as a vaccine or if it is used on an ELISA plate. The protein used to immunize is called the immunogen, and the protein on the plate is called the antigen. The source of peptides is the epitope's source antigen. We also capture in vivo and in vitro processes. The former includes natural infection, administration of a vaccine, and occurrence of disease, while the latter includes T cell restimulation.

**Figure 2:**
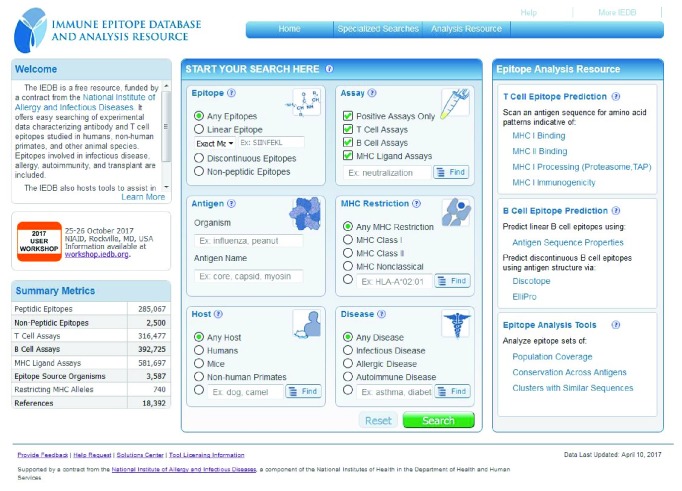
The IEDB home page features a search interface in the center of the page.

The search interface on the IEDB home page (Figure 2; http://www.iedb.org) allows performing the majority of user queries and provides the ability to refine initial queries on the subsequent results page. The home page interface contains six sections or panes that allow users to search by major categories of epitope, antigen, host, assay, MHC restriction, and disease. Clicking the green Search button brings the user to the results page. This page contains panels on the left-hand side that allow for further filtering of the results using the same six elements as before, plus a pane for references. Although some of these panes are similar to those on the home page, they allow users to be more specific in selecting search criteria and thus refining the query.

The query results appear on four different tabs. The Epitope tab lists the epitope description or sequence, as well as the antigen and organism from which it is derived. The Antigen tab contains the antigen name and corresponding organism. The Assay tab summarizes information for the T cell, B cell, and MHC ligand responses and respective journal articles. The Reference tab lists all the papers and submissions providing results for the query. The number of records in each tab is listed in parentheses. Users can sort results on these tabs by clicking on the column header. The results of each tab can be downloaded as comma-separated value (CSV) files by clicking on the Excel icon. They can then be opened by a spreadsheet program for further analysis and data manipulation.

All queries are run with positive assay results as the default because most people expect this behavior. However, it is worth stressing that the IEDB also captures negative experimental results. By making use of this information, researchers can decide not to repeat experiments that others have conducted. These data points are also important in training machine learning algorithms.

To simplify and at the same time ensure rigor in definitions and data searches, behind the scene, we make extensive use of ontological terminology, such as “parent-child” relationships. For example, the protein hemagglutinin has different hemagglutinin children that represent that protein for specific strains. The relationships between the epitope, the protein from which it is derived, and the organism that is the source of the protein are all captured. Further description of the terms and nomenclature can be found on the Data Field Descriptions page (http://curationwiki.iedb.org/wiki/index.php/Data_Field_Descriptions) and in the IEDB Curation Manual (http://curationwiki.iedb.org/wiki/index.php/Curation_Manual2.0).

## 7. Easy Options to Narrow Search Results

As a user moves the mouse over each search pane on the results page, it expands to reveal free-text fields and finder buttons. In the Epitope pane, by default the radio button for “Any Epitopes” is selected, but users can restrict their search to linear, discontinuous, or nonpeptidic epitopes. By expanding this pane, users can specify a sequence for a linear epitope. They can also select whether they want an exact match to the sequence, find it as a substring in another epitope, or find epitopes that are homologous to it at 70%, 80%, or 90% sequence identity. A specific finder enables the selection of a nonpeptidic structure.

The Antigen pane has two fields, Organism and Antigen Name, which permit users to search for pathogen, allergen, or self-antigen from which the epitope was derived. For autoimmune diseases, these would be human proteins, and for allergens, these would include sources such as grass, pollen, or dust mites. The text fields are auto-complete enabled, so as the user starts typing, possible values will be listed below to help speed the input process. Synonyms are included, so typing HCV, for example, will produce Hepatitis C virus as an option.

The Assay pane by default selects all assay types that have positive measurements. The search can be refined by deselecting these options and by specifying assay types with the auto-complete text fields or finders for the three major assay types—T cell, B cell, and MHC ligand (binding and elution).

The MHC Restriction pane enables queries for the restrictions captured for a given epitope in a given T cell, MHC binding, or elution assay. Users can constrain the search at a high level to class I, class II, or nonclassical broad categories. They can also specify one or more alleles using the auto-complete text field or the finder.

The Host pane lets users define the host in which the epitope was described. The Host pane on the home page has selections for Any, Humans, Mice, Non-human Primates, and a text field for specifying other curated host species. On the results page, the Host pane replaces the Other Common Hosts with a Specific Host option with an accompanying auto-complete field and finder. This allows the specification of transgenic mice or particular mouse strains.

The Disease pane enables searching for data based on the clinical status of the host as detailed in the patient history or for a known animal model disease, such as experimental allergic encephalomyelitis (EAE) for multiple sclerosis. As such, it makes searching for autoimmune and allergens easier since antigens in these disease categories are not necessarily pathogenomonic, as they are with infectious disease antigens. Users can specify diseases with the auto-complete text field or the associated finder. Finally, the Reference pane can be used to limit searches to journal articles or submissions, if desired, or by author, title, year, or PubMed ID. In general, users can also specify author, reference titles, and year.

## 8. Using the Antigen, Organism, MHC Allele, Assay, and Disease Finders

As described above, finders provide standardization and hierarchical organization of the data in the IEDB and help the users formulate search criteria at different levels of granularity. The tree structure graphically displays parent-child relationships of the data. Finders exist for antigens, nonpeptidic molecules, organisms, MHC alleles, assays, and disease.

The Epitope pane contains a finder for nonprotein chemical entities, including metal allergens, such as nickel; drug compounds, such as penicillin; and glycolipids, such as lipopolysaccharides. The Antigen pane also contains an antigen finder for specifying the molecular source of the epitope (in most cases a protein). For example, a user interested in hemagglutinin for influenza A virus can type “HA” in the Name field and select “influenza A” using the auto-complete feature in the Source Organism text field and then click Search to generate a list of potential hemagglutinin hits.

**Figure 3:**
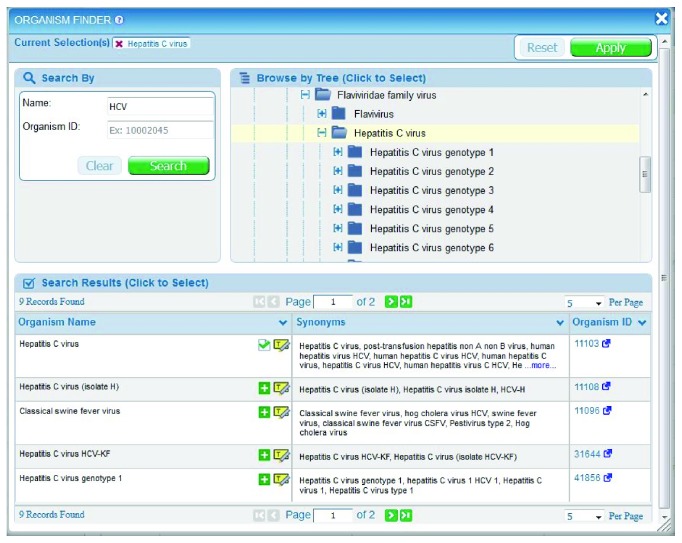
The Organism Finder can be used to select the search criteria for the epitope source organism. A similar finder is available for selecting the host organism.

As mentioned above, the organism finder is used to specify the source of the epitope or the assay immunogen or antigen. To do this, users can open the finder and type “HCV” in the Name text field in the upper left corner and click the Search button on the finder. This generates a list of related terms below. Users can then click on the green “+” icon to make a choice, which then appears at the top of the finder in Current Selections at top. Alternatively, a user can click on the yellow highlight icon, which will highlight the selected organism in the tree structure in the center (Figure 3). This highlight feature enables the user to visualize the taxonomic relationships and allows the user to select a strain, species, genus, or family. In either case, the user must click the green Apply button to complete the organism specification for that query. The organism finder can be accessed by the Antigen pane.

The Assay pane contains the assay finder that is used to find specific assay types within the broader category of B cell, T cell, or MHC ligand. Users can search by name, method, or “measurement of.” In this way, users can specify assay methods such as ELISA, ELISPOT, ICS, or X-ray crystallography or can alternatively specify all assays that measure interferon gamma (IFN*γ*).

The allele finder in the Allele pane is used to specify the MHC restriction defined in the assay. As of January 2017, the IEDB MHC tree contains alleles from 16 different species, including primarily human, nonhuman primates, and mice. Users can type in the name of their alleles of interest in the Name text field or use the pull-down menus for Organism or Class and then click the Search button to generate a list of possible alleles. As with the other finders, users can select from the list or highlight an allele to expand the allele tree. The use of the finder is especially convenient since the allele notation can change over time.

The host finder on the Host pane allows users to select additional host species that are not available from the Host pane on the home page. This might include specific mouse strains, such as transgenic mouse species with complex names.

As discussed above, the disease finder within the Disease pane lets the user specify a disease, which is helpful when searching for autoimmune diseases or allergens. For example, a search for “asthma” using the Disease Name text field finds two types of asthma, allergic asthma and nonallergic/occupational asthma. Again, it should be emphasized that this field does not search for epitope data based on disease category but rather by the clinical state of the subject or animal model.

## 9. Specialized Search Options

While the home page search interface can accommodate the vast majority of common searches, more intricate or complex immunological queries may need one of the specialized search interfaces. The specialized searches can be accessed from the home page under the Specialized Searches pull-down menu. The first four items in the list, Epitope Details, T Cell Assay Details, B Cell Assay Details, and MHC Assay Details, allow the user to access every field in the database, greatly expanding the search options.

The first Specialized Search in the menu is Epitope Details. This page has a similar layout to the Results page in the home page search interface. It has the same four results tabs, but the filter selections on the left are different and can be expanded to reveal numerous fields that were not previously available. The Epitope Reference Details section contains fields for epitope name and start/stop positions in a reference protein. The Epitope Related Object section includes fields intended to capture entities that bear some relationship to the epitope—analog, mimotope, and neoepitope. Analogs are synthetic constructs of peptide sequences or chemical compounds that share some structural features in common with another sequence or compound. They are often used to determine the role of specific amino acids in the binding or immunogenicity of an epitope. The source of an analog is always artificial. Mimotopes are functional mimics of natural molecular structures which bear little or no sequence homology to their biological counterparts. Cancer neoepitopes are defined herein as any epitope comprising amino acids that are not encoded in the germ line genome but that arise due to somatic (nonsynonomous) mutations, excluding somatic rearrangements of TCR and Ig genes. The IEDB will curate both immunogenic neoepitopes and putative epitopes tested for recognition that were not found to be immunogenic. Users can search by author, keywords in abstracts, journal name, MeSH terms, year, and more.

The T Cell, B Cell, and MHC assay details pages all have the same capabilities described above plus two others—Host and Assay. The Host pane contains fields related to how the host acquired immune reactivity, such as being immunized or by natural infection. Users can also specify the immunogen (e.g., epitope, protein, or organism) and the host disease, among many other criteria. In the Assay pane, in addition to selecting assay type, users can specify effector cells, antibody isotypes, monoclonal or polyclonal response, and the assay antigen (e.g., epitope, protein, or organism).

The Host pane also has data fields for the first and second in vivo processes. An in vivo process indicates how the host acquired immune reactivity, such as by immunization or natural exposure. A host can also encounter an immunogen more than once, which is why two in vivo processes are available. For example, a mouse can be immunized with a peptide and then later challenged with an organism and the cells taken from the organism and assayed in vitro. That scenario is captured with two in vivo processes, the first for the immunization of the mouse and the second for the challenge. There are also fields to capture in vitro process because often there is a restimulation performed in vitro prior to the assay.

To specify in vivo processes, the Host pane contains a multiselect pull-down menu with a variety of choices that can be categorized into groups related to administration, occurrence, exposure, transplant/transfusion, no immunization, and unknown. Definitions for all the choices are provided in the IEDB Curation Manual. The 1st Immunogen section has fields to extend the criteria of the 1st In Vivo Process. Users can indicate the relationship of the immunogen to the epitope. For example, should the immunogen be the epitope itself, the source antigen of the epitope, or have a taxonomic relationship to the epitope, such as an epitope that is reported from strain A, but the immunogen used is from strain B. These fields are mirrored in the antigen and assay fields as well.

The final specialized search is the Identifier Search, which is divided into two sections. The top section has query fields for internal IEDB identifiers for Epitope ID, Reference ID, Submission ID, and Assay ID. All curated articles and submission are given a Reference ID. In addition, data submissions are given a Submission ID that researchers can use in their publications. When the paper is published and curated, a link in the database is created between the paper and corresponding submission. All assays and epitopes are also assigned unique identifiers. The bottom section of the Identifier Search contains query fields for identifiers assigned by three external databases that have links to the IEDB. These databases are PubMed, the Protein Data Bank (http://www.rcsb.org), and ChEBI.

## 10. The Immunome Browser Reporting Tool

The Immunome Browser (IB) is a unique analysis tool that is integrated into the query interface results page to visualize query results [17]. It displays all T cell or antibody responses along an individual protein or polyprotein/proteome. It is useful for visualizing dominant epitope regions and indicating which regions of an antigen are experimentally well-characterized. In general, it is difficult to say what an immunodominant epitope is because it is content-dependent. The IB uses host response frequency data, which is the ratio of the number of respondents to the number of subjects tested, to give an indication of the overall epitope prominence. This response frequency score (RFscore) is mapped on a residue basis onto a reference protein or proteome obtained from NCBI.

HCV provides an excellent example to demonstrate the utility of this tool because its genomic polyprotein is about 3000 amino acids in length, yet there are more than 4000 HCV epitopes reported from the literature and therefore captured in the IEDB [17]. Though seemingly counterintuitive, the large number of epitopes is explained by the fact that many variants of HCV sequences in overlapping, but not identical frames, have been tested in hundreds of different assays (T and B cell) in multiple host systems. To start, the user types “HCV” in the Organism text field in the Antigen pane on the home page. The auto-complete feature presents a list of possibilities, and the user can select the top item, Hepatitis C virus (ID:11103). Upon clicking the Search button, approximately 4500 epitopes, over 13,000 assays, and nearly 600 references are returned in the Results page. The IB is accessed from the Antigen tab. When this tab is viewed for the first time, a pop-up help window appears that points to the IB icon and states that the “Immunome Browser maps epitopes retrieved from a query onto their source protein to visualize how often different regions in a protein have been tested and how often they were positive.”

**Figure 4:**
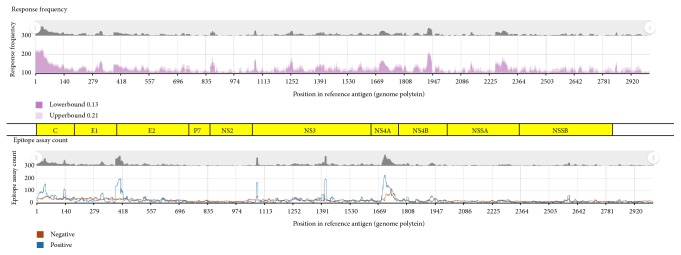
The Immunome Browser maps response frequencies and assay counts onto a reference antigen. The polyprotein for HCV is superimposed on the plots for reference. The data reflects IEDB HCV content as of October 2016.

Clicking on the IB icon for the HCV genomic polyprotein generates the IB page, which contains two graphs and a data table. The top graph maps the response frequency, starting at residue 1 and extending to residue 3011.  Figure 4 shows the response frequency with an overlay of the individual proteins that make up the HCV genome. There is a large response for the core protein at the N-terminus and good responses for the NS4B and NS5A regions. The graph plots two lines, one in light pink and the other in dark pink. These denote the upper and lower bound, respectively, of the 95% confidence interval. The closer these two lines are to one another, the greater the confidence in the response at that residue position. Each IB plot of response frequency scores will automatically include the name if the reference proteome/genome selected for mapping of queried epitope data; however, the graphical overlay of the individual HCV proteins depicted here was superimposed within the figure as a point of reference and is not an automated feature of the IB.

**Figure 5:**
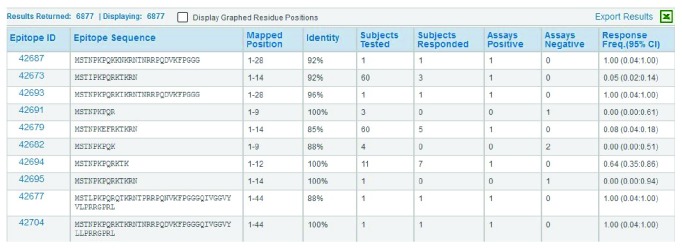
The Immunome Browser generates a table that lists all epitopes and corresponding response information sorted by their mapped position. This table can be downloaded in a CSV-formatted file by clicking on the “Export Results” link in the upper right corner.

The bottom graph plots the positive and negative assay counts, which is helpful in interpreting the upper graph. A region of low response frequency might reflect negative assay results or the fact that few experiments have covered that portion of the genome. The data table at the bottom of the web page (Figure 5) lists all the epitopes by position and their related data. The table contains columns for the response frequency, the number of subjects tested and responded, and the number of positive and negative assays. This table can be downloaded into Excel or other spreadsheet programs for further analysis, for example, sorting on epitopes with high RFscores.

## 11. An Example Query Using the Home Page Search Interface

**Figure 6:**
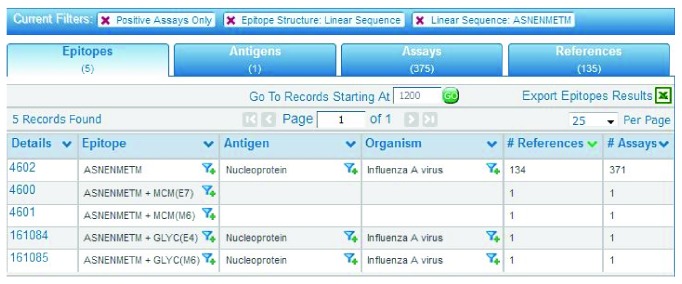
The results for a home page query for the linear sequence ASNENMETM, a well-characterized epitope of influenza A virus nucleoprotein. The results include four epitopes with posttranslational modifications.

Here, we use as an example the well-characterized epitope from influenza A nucleoprotein (NP) protein, ASNENMETM. This can be done by entering or pasting the sequence in the Epitope pane sequence text field on the home page and clicking the Search button. As of January 2017, performing this query results in five epitopes, including the naturally occurring sequence from influenza A virus, and four entries representing that epitope with different posttranslational modifications. One of them, ASNENMETM + MCM(E7), indicates the main chain modification of residue seven. Another, ASNENMETM + GLY(E4), indicates a glycosylation of residue four. The sequence was identified in 375 different assays described in 135 references, as listed on the Assay and Reference tabs, respectively, as seen in Figure 6. The Assay tab itself has three tabs for T cell, B cell, and MHC Ligand assays (binding and elution). Most of the assays from this query are T cell (325), with the others being MHC ligand assays (50). Each row in the Assays tab result table represents the elements for one assay. The columns indicate the reference, epitope, host, immunization process, the assayed antigen, its relationship to the epitope, the MHC restriction (if defined and recorded), the assay type, and the qualitative value (positive or negative). By clicking on the Assay ID in the first column, users can access the relevant Assay Details page, in order to see the full curation. The Reference tab provides a summary of the articles and submissions that were curated. As mentioned above, the Ref ID is a unique internal identifier assigned by the IEDB to journal articles and data submissions. There is also a column for PubMed IDs, which is linked to the relevant entry in PubMed.

On the results page, users can take advantage of the various filter functions embedded throughout the tabs. For example, each epitope record in the Epitopes tab has a funnel icon to the right of the sequence. Clicking on this icon redisplays the query results to show data only for that epitope. In the case of the epitope ASNENMETM with Epitope ID 4602, clicking on the filter icon generates results with only one epitope, one antigen, 371 assays, and 134 references, slightly less data than the original query since the posttranslational modifications are omitted.

Users can also search for epitopes that are similar. On the home page, in addition to typing “ASNENMETM” in the Epitope pane text field, the user can use the pull-down menu to the left of the text field to select three different levels (70%, 80%, and 90%) for a BLAST match of the sequence. Setting the level to 70% and clicking the Search button for ASNENMETM yields 176 epitopes. Many of these are naturally occurring and are variations of the nucleoprotein (NP) of influenza A virus from different strains. The results table in Figure 6 also contains epitopes with no antigen or organism listed. These are termed analogs as their amino acid sequence does not have a natural source. By clicking on the filter icon for one of these analogs, Epitope ID 733, and then viewing the Assays tab of the new results, the user sees there are four assays, two T cell, and two MHC ligand. The user can find more information about each assay by clicking on the Assay ID in the leftmost column. The Assay details page contains all the information on that particular experiment, including reference (journal article or data submission), epitope, host, immunization, assay type, and antigen. In the case of Assay ID 1004030, the details page states that it is an analog in the Related Object Type row. Other details include describing the assay as a chromium release, the host as a C57BL/6 mouse, the in vitro administration as involving restimulation in vitro with effector cells from the spleen, and the antigen presenting cells as EL-4.

## 12. Examples That Highlight Additional Query and Reporting Capabilities

The first example provides insight into the utility of downloading query results with the Excel export option, using a query for all T cell epitopes for Dengue virus. It can be executed from the home page by typing “dengue” in the Antigen pane's Organism text field and selecting “Dengue virus (ID: 12637)” from the auto-complete list, and then by unchecking the B Cell Assays and MHC Ligand Assays boxes in the Assay pane before clicking the Search button. As of January 2017, this query yields 1940 epitopes, 2 antigens, 3506 assays, and 85 references in the results set. Going to the Assay tab, the user can click the Excel icon to download data and further analyze it. The download of data from the Assays tab will provide a complete set of data, including details for the epitope, immunization processes, immunogens, antigens, all assay types, and reference data. The Assay tab itself contains three subtabs, one for each general assay type, and the assay count is listed at the top of the tab in parentheses.

The downloaded file is in a comma-separated value (CSV) format that can be opened in Excel or other spreadsheet programs. It contains numerous columns, each one representing a data field. The first nine columns describe the reference, followed by twelve columns describing the epitope. Other groups include host, in vivo processes, assay, antigen, MHC allele, and more. Users can sort and filter the data and utilize a variety of Excel functions to manipulate the data and perform analyses.

The second example involves using the protein tree within the Antigen finder to query for envelope proteins of several types of *Flavivirus*. Clicking on the Search button on the home page search interface leads to the results page where the antigen finder can be accessed in the Antigen pane. Clicking the blue Finder button next to Antigen Name opens this feature. By typing “flavivirus” in the Source Organism text field and selecting “Flavivirus (ID: 11051)” from the auto-complete list and clicking the Search button returns search results that appear in the bottom section of the finder. Clicking the yellow highlight icon next to “Flavivirus protein” expands the protein node of the molecule finder tree. In this case, family Flaviviridae is a high node on the tree and the first branch is Flavivirus protein representing a genus under that node. Branches within that node represent proteins from different Flavivirus species (dengue, JEV, etc.). The user can expand the Dengue virus protein nodes to reveal the envelope protein node and repeat this process to select envelope proteins from Japanese encephalitis virus, West Nile virus, Yellow fever virus, and so on, to examine responses to this protein across species. The results can also be visualized in the Immunome Browser.

## 13. Example Queries Using Specialized Searches

The specialized search interfaces enable more complex and intricate queries than can be performed with the home page search interface. For example, the T Cell Details Specialized search can be used to find Rift Valley fever virus T cell epitopes testing specifically on CD8^+^ T cells and defined in humans. This search may specifically query for experiments where the authors purify and test CD8^+^ T cells. To perform this search, the user would select T Cell Assays Details from the Specialized Searches pull-down menu and then perform the following actions. In the Epitope pane on the left, type “rift” in the Organism text field and select “Rift Valley fever virus (ID: 11588)” from the auto-complete list. In the Host pane, type “human” in the Host Organism text field and select “Homo sapiens (human) (ID: 9606, human)” from the auto-complete list. In the Assay pane, expand the Effector Cells section and select “T cell CD8+” from the multiselect pull-down menu for Effect Cell Type. Upon clicking the Search button, the results are displayed in the same format as appears for the home page search, showing 61 epitopes and 73 assays.

A user might want to further refine the query results to find out if those CD8+ T cell epitopes have been shown to work as tetramers or if tetramers have been made and shown to be effective. The query above can be refined by typing “tetramer” in the Assay text field in the Assay pane and selecting “qualitative binding/multimer/tetramer” from the auto-complete list. The Search button on the page will have changed from grey to green to indicate that it must be clicked to reinitialize the search. The refined search results show that the IEDB contains two epitopes and two positive tetramer assays.

The second specialized search example uses the B Cell Details search to find Influenza A virus epitopes recognized by neutralizing human monoclonal antibodies. Starting with the B Cell Details search page, the user would specify organism as Influenza A in the Epitope pane and host organism as human in the Host pane. For the specialized searches, the qualitative assay is not set to positive by default, so it would be necessary to select “positive” in the pull-down menu in the Assay pane for Qualitative Measurement. Next, the user would perform the following actions: in the same pane, type “neutralization” in the Assay text field and select “neutralization|biological activity” from the auto-complete list. To specify monoclonal antibodies, expand the Assayed Antibody section on the Assay pane and select “Monoclonal” from the drop-down multiselect list under Antibody Purification Status. Other choices in this menu include polyclonal and display library. Clicking the Search button reveals 66 epitopes and 575 B cell assays.

## 14. Example Queries for DENV Monoclonal Antibodies, Influenza Tetramer Assays, and RSV Protective Antibodies

The next query example is what monoclonal antibodies have been described for Dengue virus, which requires the B cell assay specialized search. Here, the user would perform the following actions: in the Assay pane, expand “Assay Antibody,” open the menu for Antibody Purification Status, and check “monoclonal.” “Display Library (monoclonal)” can also be selected if desired. In the Epitope pane, type “dengue” in the Organism text box and click on “Dengue virus (ID: 12637),” and then click the Search button to generate a list of all monoclonal antibodies for Dengue. To further explore the results, users can go to the Assay tab and download the results in an Excel-readable file. Once in Excel, users can easily sort and filter data. For example, users can filter on the Assay/Assay Group column for neutralization assays and further filter on the 1st in vivo Process column for Occurrence of Natural Infection. In addition, information is available on isotype and other assay variables. If users are interested in 3-dimensional structures, they can open the Assay finder in the Assay pane and click on “3D structure.” If users are interested in a particular PDB ID, they can search for it by expanding the 3D Structure of Complex in the Assay pane and enter the PDB ID in the corresponding text field.

Other example queries for tetramers are used in the context of Influenza A virus and associated MHC alleles. To specify an assay type, the user will need to access an assay finder. Starting on the home page, the user immediately clicks the Search button to get to the Results page. In the Organism text field in the Antigen pane, the user would type “influenza” and the auto-complete function will list “Influenza A virus (ID: 11320).” Next, the user would perform the following actions: select this value and then deselect B Cell Assays and MHC Ligand Assays in the Assay pane. In that same pane, type “tetramer” in the T Cell Assays text field. From the auto-complete-generated list, select “qualitative binding|multimer/tetramer” assay and click the Search button. This will produce a list of over 400 epitopes. The user can view the alleles on the Assays tab in the MHC Restriction column, which is sortable. Users can also download the results of the Assay tab and find the allele information in the MHC/Allele Name column in one of the far right columns in the spreadsheet. There is also a column for Effector Cells/Cell Type that can be sorted and filtered.

The final infectious disease query example involves finding protective antibody epitopes for respiratory syncytial virus (RSV). This search involves targeting a specific type of assay for a pathogen of interest. Thus, from the home page, the user clicks the Search button to get to the filters on the Results page. In the Antigen pane, typing “RSV” will generate an auto-complete list with numerous related individual species choices. Thus, in order to select a high node including all RSVs, the user can instead open the finder and type “rsv” in the organism name text box. Once done, the user can click on the yellow highlight icon in one of the rows to open up the organism tree to reveal bovine RSV, human RSV, and other related organisms under the Orthopneumovirus node. From here, the user can select this higher node and click Apply. The user next specifies the assay type in the finder in the Assay pane by typing “challenge” in the name field and clicking Search to get a list of challenge assays. By clicking on the highlight icon in one of the finder search result rows, the assay tree is opened to reveal the hierarchical structure. Next, the user would perform the following actions: select the “challenge” node, click Apply, and then click Search on the Results page to reveal at least 17 epitopes in the context of an in vivo challenge assay where, in most instances, an animal model was challenged with live virus to demonstrate whether or not an epitope could elicit protective immunity.

## 15. Example Queries for Human Diabetes Epitopes

The first query seeks to specifically identify all human data available for type 1 diabetes (T1D), including what antigens have been mapped for T cell epitopes, and how many therapeutic epitopes have been identified. The query can be performed with the home page search by clicking Search to get to the Results page and then perform the following actions: in the Disease pane, type “diabetes” in the Specific Disease text field and select “diabetes mellitus” from the auto-complete list. In the Host pane, select the “human” radio button and in the Assay pane, uncheck B Cell Assays and MHC Ligand Assays, and click the Search button. The results of the search show over 450 epitopes. The Antigens tab displays the antigens in descending order of the number of epitopes. In this case, Gluctamate decarboxylase 2 (GAD), one of the predominant antigens for type 1 diabetes, appears at the top of the list.

Next, to find the subset of these data that is associated with treatments or decreased disease, the user can refine the initial search by adding the criteria of T cell assays related to treatment. In the T Cell Assay text field, type “treatment” and select “decreased disease in vivo assay (reduction of disease after treatment)” and click the Search button. As of January 2017, this search revealed two epitopes found to be therapeutic in humans with T1D. The final example query builds on the previous one, asking if there are immunodominant regions defined for insulin in humans with T1D. Perform the query as above for humans, T1D and T cell responses. Then, on the antigen tab, click the Immunome Browser icon next to insulin. This will display the response frequency of all assays mapped onto a reference antigen for insulin. From this, the user can visualize the immunological hot spots.

## 16. Conclusions

The IEDB provides biomedical researchers interested in the development of vaccines and therapeutics a unique resource for epitopes related to a wide variety of infectious diseases, allergens, autoimmune diseases, and alloantigens. The database content of peptidic, conformational, and nonpeptidic epitopes and their associated T cell, B cell, MHC binding, and MHC ligand elution assays is kept current with the scientific literature and includes data depositions from the research community. All data in the IEDB are experimentally derived, and it contains both positive and negative data, an important consideration for experiment design and for those interested in developing machine learning algorithms for predicting epitopes.

The IEDB has certain constraints and limitations. The IEDB does not curate HIV or cancer references, as these data are not within the programmatic scope of the NIAID. However, exceptions for the inclusion of HIV and cancer epitopes are made if they are reported alongside other epitopes. Similarly, any epitope reported in the context of a cancer resulting from viral infection (e.g., human papilloma virus and human T-lymphotropic virus) are included. The database is also limited by the information provided in each paper by the authors. For example, the type of disease (disease state) is captured in accordance with the patient histories provided by the author. The IEDB primarily describes disease states using the Disease Ontology (DO), which uses formal nomenclature and synonyms. The progress of the disease (disease stage) is described using a searchable list of terms, such as acute, chronic, and post.

There are several user interfaces that researchers can use to query the database. The home page search interface features six query criteria used in most searches. Search results can be further refined with additional filters, similar to the search paradigm used on most travel websites and many shopping websites. The results page includes four separate tabs that list epitopes, antigens, assays, and references (journal articles and data submissions). A visualization of the results mapped on to a reference proteome called the Immunome Browser can be accessed from the Antigen tab. Users can also access all the fields in the data schema with the specialized search interfaces. These interfaces enable database queries that would not be possible via the home page interface. All results can be downloaded as a CSV file for further processing in a spreadsheet application. Examples are presented to illustrate the features of the home page and specialized search interfaces for a variety of applications, including infectious and autoimmune diseases.
